# Supplementary material for: A Gram Scale Soft-Template Synthesis of Heteroatom Doped Nanoporous Hollow Carbon Spheres for Oxygen Reduction Reaction
Source: Nanomaterials (Basel). 2023 Sep 13;13(18):2555. doi: 10.3390/nano13182555 (PMC10534767; doi:10.3390/nano13182555)
Supplement: Supplementary file 1 [file nanomaterials-13-02555-s001.zip › nanomaterials-2563115-supplementary.pdf]

Supplementary

Evaluation of Electrochemical Properties

In this study, the performance of Co-N-HNCS for the oxygen reduction reaction (ORR) was investigated using a half-cell system consisting of a spinning disk electrode and potentiostat (VSP; BioLogic, France) in a three-electrode configuration. A glassy carbon electrode (GC, 5 mm; Pine, USA) with a surface area of 0.196 cm^2 was used as the working electrode, while an Ag/AgCl electrode (Pine, RREF0021, saturated KCl) and a graphite rod (Alfa Aesar, 99.9995 %) were used as the reference and counter electrodes, respectively, in an acidic electrolyte. In an alkaline electrolyte, a Mercury/Mercurous Oxide Electrode (Hg/HgO, CHI, 1 M NaOH) was used as the reference electrode. The GC electrodes were polished with alumina powder (Alpha alumina powder; CH Instruments, USA) before the experiments. A carbon ink was prepared by dispersing nanoporous carbon (7.5 mg) in a mixture of absolute ethanol (1.23 mL; DAEJUNG, 99.9%) and Nafion® perfluorinated resin solution(18.8 μL; PFSA, Sigma-Aldrich, EW 1100, 10 wt.% aqueous dispersion), and applied to the GC electrode. To ensure consistency, the carbon ink was treated using a resonant acoustic mixer (Pharma RAM I; RESODYN, USA) and ultrasonication for 5 and 30 minutes, respectively. This process was repeated twice to achieve thorough dispersion. A total of 9.41 μl of the ink was applied twice to the GC electrode, resulting in a surface loading mass of 600 μg cm^{-2}.

To investigate the performance of the oxygen reduction reaction of Co-N-HNCS, two different cyclic voltammetry protocols were used. First, the working electrode was activated at specific voltage ranges and scan rates of 0.05-1.0 V (vs. RHE) for 20 cycles at 100 mV s^{-1} and under 0.05–1.0 V (vs. RHE) for 3 cycles at 50 mV s^{-1} in either 0.1 M $HClO_4$ or 0.1 M KOH electrolyte under N_2 purged. Then, measurements were taken using a scan rate of 10 mV s^{-1} between 0.05 - 1.2 V (vs. RHE) under conditions of 1600 rpm rotation and O_2 purging. The catalyst activity for ORR was assessed at a sweep rate of 10 mVs^{-1} between 1.2 - 0.1 V (vs. RHE).

To standardize the measurements, the potential was converted to the RHE (Reversible Hydrogen Electrode) for all experiments by calibrating in hydrogen-purged electrolytes.

Table S1. Specific surface area, micro, meso, and total pore volume of nanoporous carbon materials. The pore volumes of nanoporous carbon were obtained by integrating PSD data under 30 nm for interparticle porosity.

Nanoporous carbon	${S_{BET}}^{a}$ $(m^2 g^{-1})$	${V_{micro}}^{b}$ $(cm^3 g^{-1})$	${V_{meso}}^{c}$ $(cm^3 g^{-1})$	${V_p}^{d}$ $(cm^3 g^{-1})$
N–HNCS-20 mL	1250	0.3	1.0	1.3
N–HNCS-5 mL	710	0.2	0.5	0.7
N–HNCS-1 mL	1300	0.2	1.4	1.6

[a] *Specific surface area*, [b] *Micropore volume* (*under* 2 *nm*), [c] *Mesopore volume* (2 ~ 30 *nm for interparticle porosity*), *and* [d] *Total pore volume* (*under* 30 *nm for interparticle porosity of nanoporous carbon*).

Table S2. XPS N 1s spectra of nanoporous carbon materials. The data was demonstrated by deconvolution of each N 1s spectra. Peaks deconvolution of binding energies was performed with the same values (Oxidized N (402.3 eV), Quaternary N (400.7 eV), Pyrrolic N (399.3 eV), Pyridinic N (398.3 eV)).

Nanoporous carbon	$XPS\ N\ 1s\ spectra$: $Deconvoluted\ species\ percentage$ %			
	Oxidized N [402.3 eV]	Quaternary N [400.7 eV]	Pyrrolic N [399.3 eV]	Pyridinic N [398.3 eV]
N-HNCS-20 mL	10.6	51.9	18.3	19.2
N-HNCS-5 mL	9.3	54.3	11.9	24.4
N-HNCS-1 mL	7.9	51.8	18.8	21.5

Table S3. EA data of nanoporous carbon materials. All data were calculated as an average value of three times and expressed as a weight percentage of each atom.

Nanoporous carbon	$EA\ (wt.\%,\ average\ for\ three\ times)$		
	C	N	N/C ratio (%)
N-HNCS-20 mL	84.8	1.7	2.0
N-HNCS-5 mL	89.0	2.3	2.6
N-HNCS-1 mL	90.9	1.9	2.1

Table S4. Specific surface area, micro, meso, and total pore volumes of N-HNCS per solvent ratio. The pore volumes of the carbons were obtained by integrating PSD data under 30 nm for interparticle porosity.

Nanoporous carbon	${S_{BET}}^{a}$ $(m^2 g^{-1})$	${V_{micro}}^{b}$ $(cm^3 g^{-1})$	${V_{meso}}^{c}$ $(cm^3 g^{-1})$	${V_p}^{d}$ $(cm^3 g^{-1})$
200 : 100	1250	0.29	1.0	1.3
200 : 80	1200	0.27	0.9	1.2
200 : 60	1400	0.30	1.2	1.5

[a] *Specific surface area*, [b] *Micropore volume* (*under* 2 *nm*), [c] *Mesopore volume* (2 ~ 30 *nm for interparticle porosity*), *and* [d] *Total pore volume* (*under* 30 *nm for interparticle porosity of nanoporous carbon*).

Table S5. XPS N 1s spectra of nanoporous carbon materials per solvent ratio. The data was demonstrated by deconvolution of each N 1s spectra. Peaks deconvolution of binding energies was performed with the same values (Oxidized N (402.3 eV), Quaternary N (400.7 eV), Pyrrolic N (399.3 eV), Pyridinic N (398.3 eV))

Nanoporous Carbon	*XPS N 1s spectra: Deconvoluted species percentage %*			
	Oxidized N [402.3 eV]	Quaternary N [400.7 eV]	Pyrrolic N [399.3 eV]	Pyridinic N [398.3 eV]
200 : 100	10.6	51.9	18.3	19.2
200 : 80	9.3	58.4	16.0	16.3
200 : 60	11.3	48.4	29.5	10.8

Table S6. EA data of nanoporous carbon materials per solvent ratio. All data were calculated as an average value of three times and expressed as a weight percentage of each atom.

Nanoporous Carbon	$EA\ (wt.\%,\ average\ for\ three\ times)$		
	C	N	N/C ratio (%)
200 : 100	90.9	1.9	2.1
200 : 80	90.6	1.95	2.1
200 : 60	90.4	1.75	1.9

Table S7. EA data of Co-N-HNCS variation samples

Nanoporous Carbon	$EA\ (wt.\%,\ average\ for\ three\ times)$		
	C	N	N/C ratio (%)
Co-N-HNCS	86.2	2.2	2.6
Co-N-HNCS-8HQ	90.2	1.6	1.9
Co-N-HNCS-1,10P	86.1	2.3	2.7
Co-N-HNCS-none	90.0	1.3	1.4

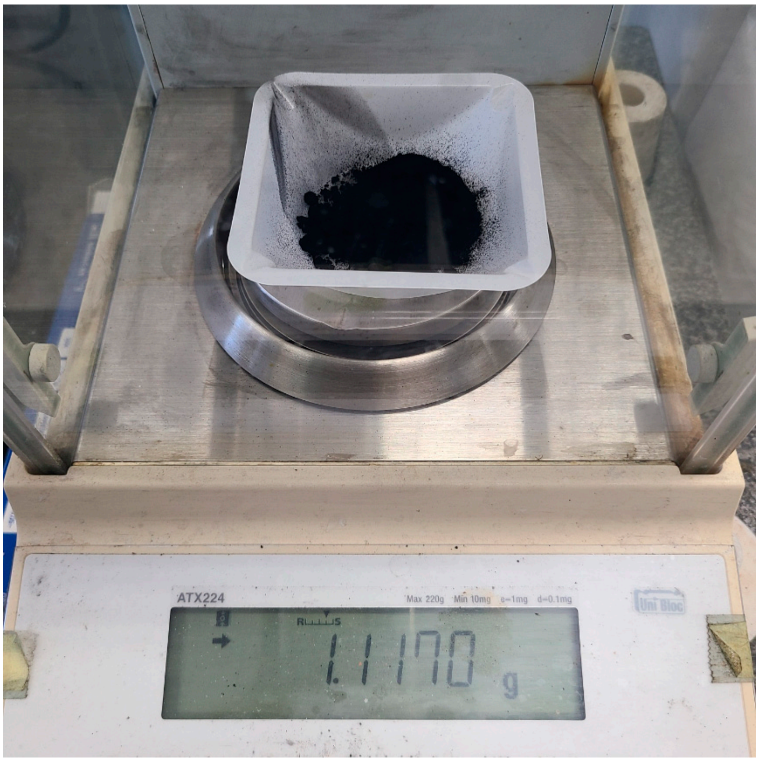

Figure S1. Picture of N-HNCS-1 and its weight

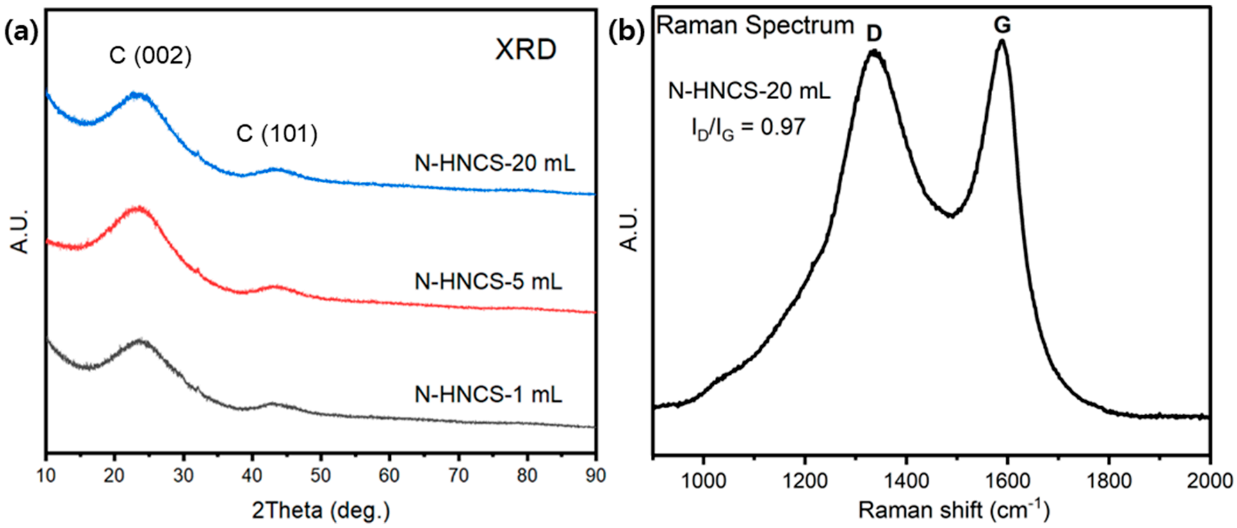

Figure S2. (a) XRD patterns of N-HNCS-1, 5, 20 mL and (b) Raman spectrum of N-HNCS-20 mL

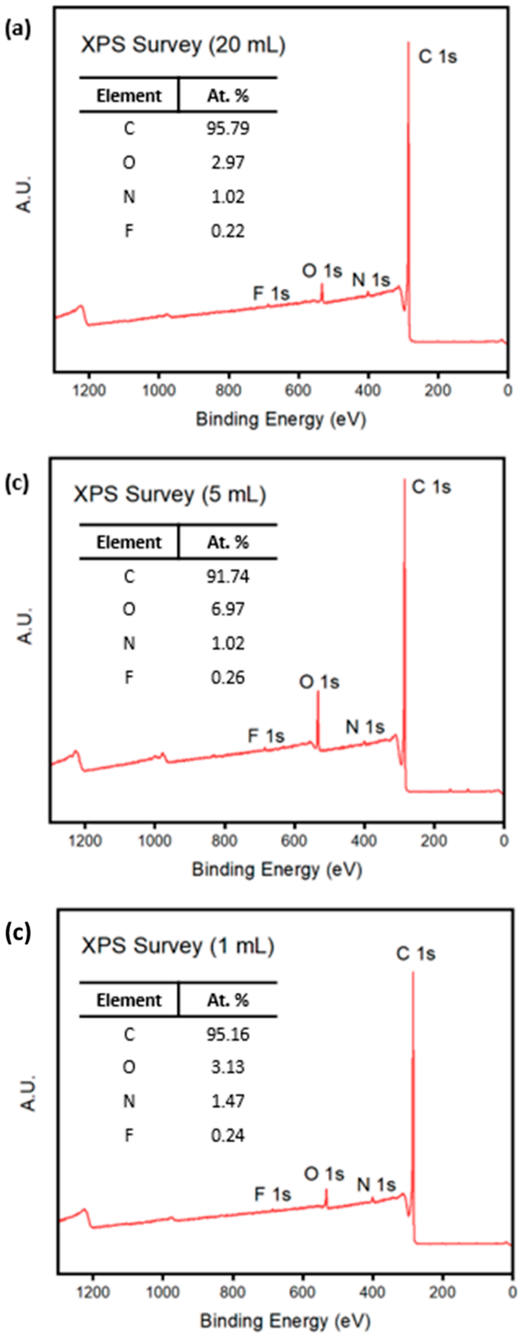

Figure S3. XPS surveys of nanoporous carbon materials: (a) N-HNCS-20 mL, (b) N-HNCS-5 mL, and (c) N-HNCS- 1mL.

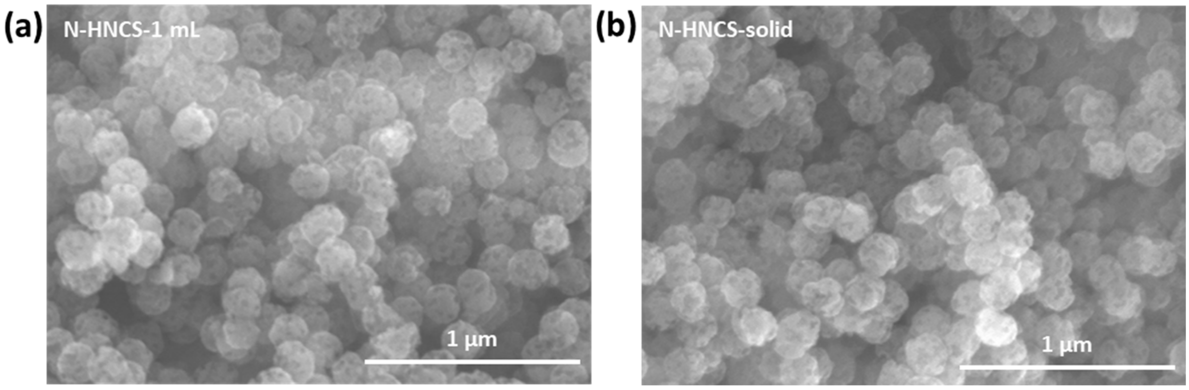

Figure S4. SEM images of N-HNCS-1 and N-HNCS-solid.

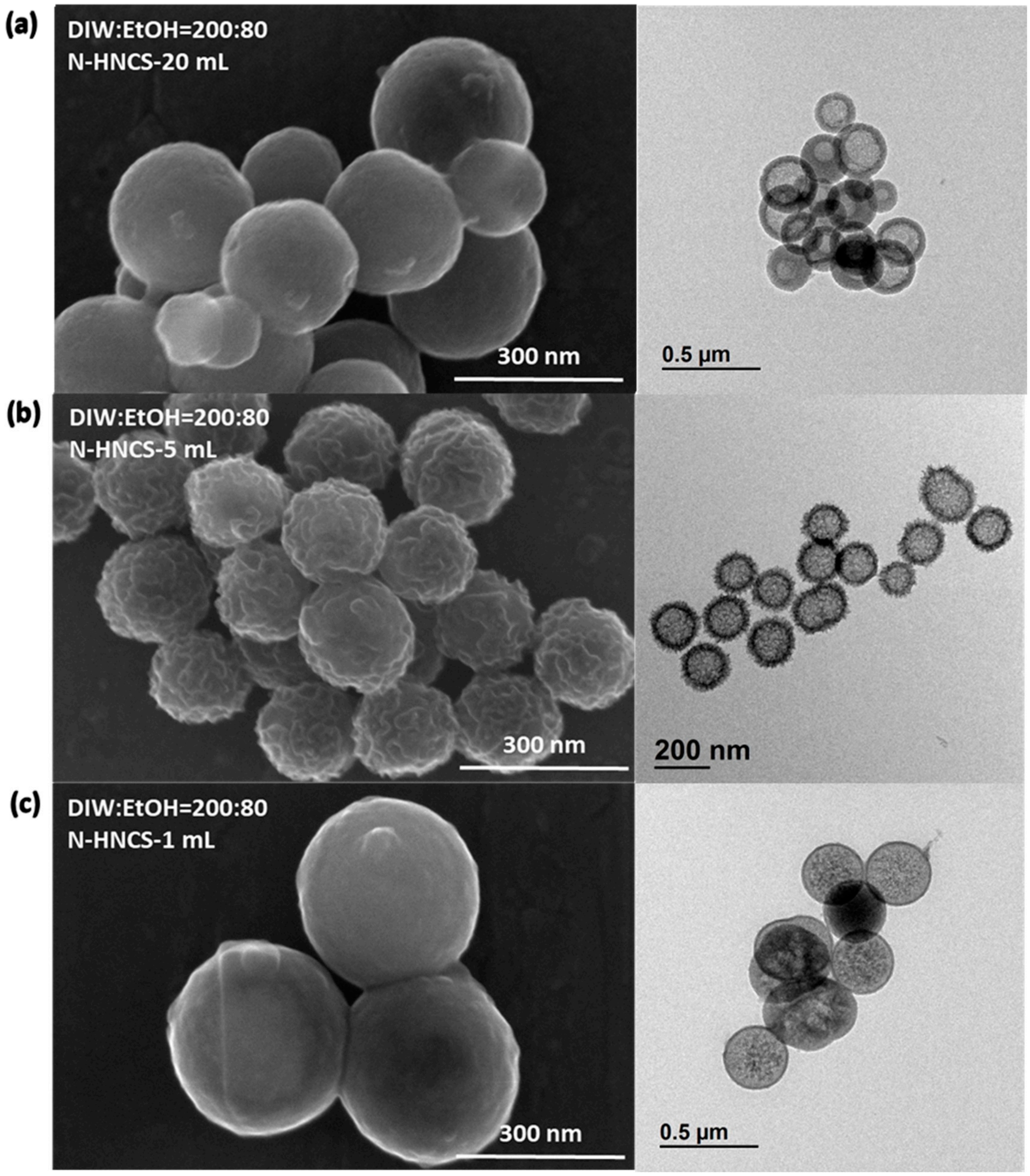

Figure S5. SEM, TEM images of N-HNCS synthesized at DIW:EtOH=200:100 mL ratios per resorcinol solution concentration. (a) 20 mL, (b) 5 mL, (c) 1 mL.

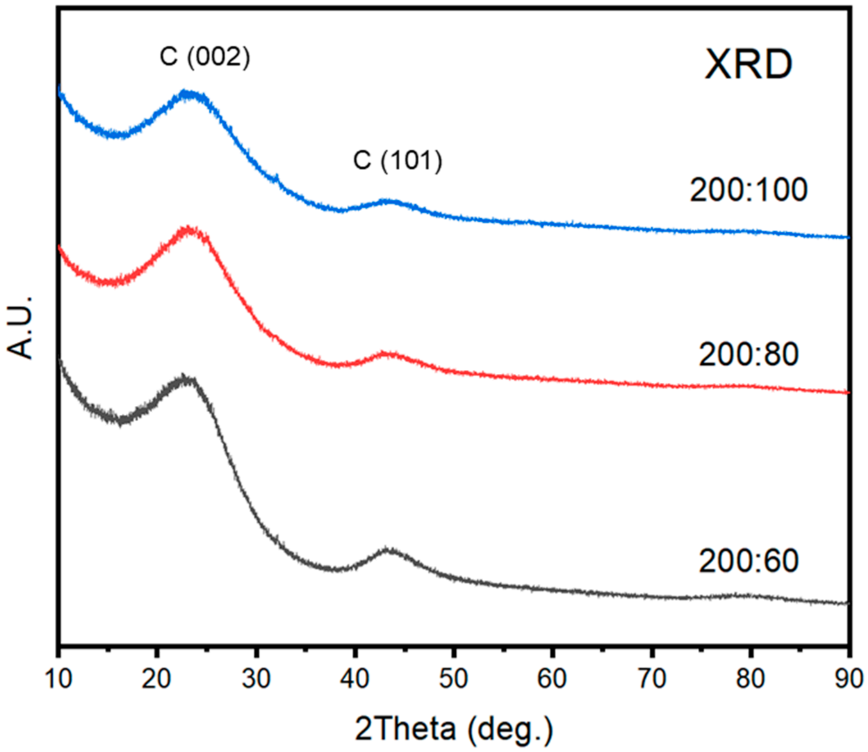

Figure S6. XRD patterns of N-HNCS per solvent ratio.

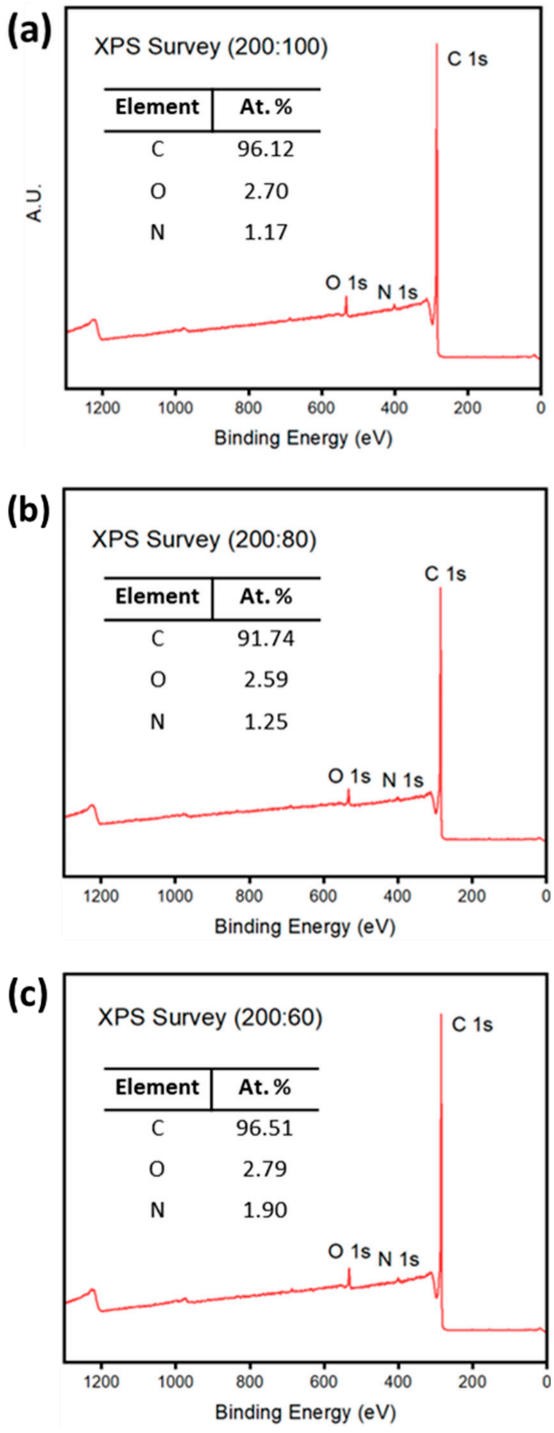

Figure S7. XPS surveys of nanoporous carbon materials: (a), 200:100, (b) 200:80, and (c) 200:60.

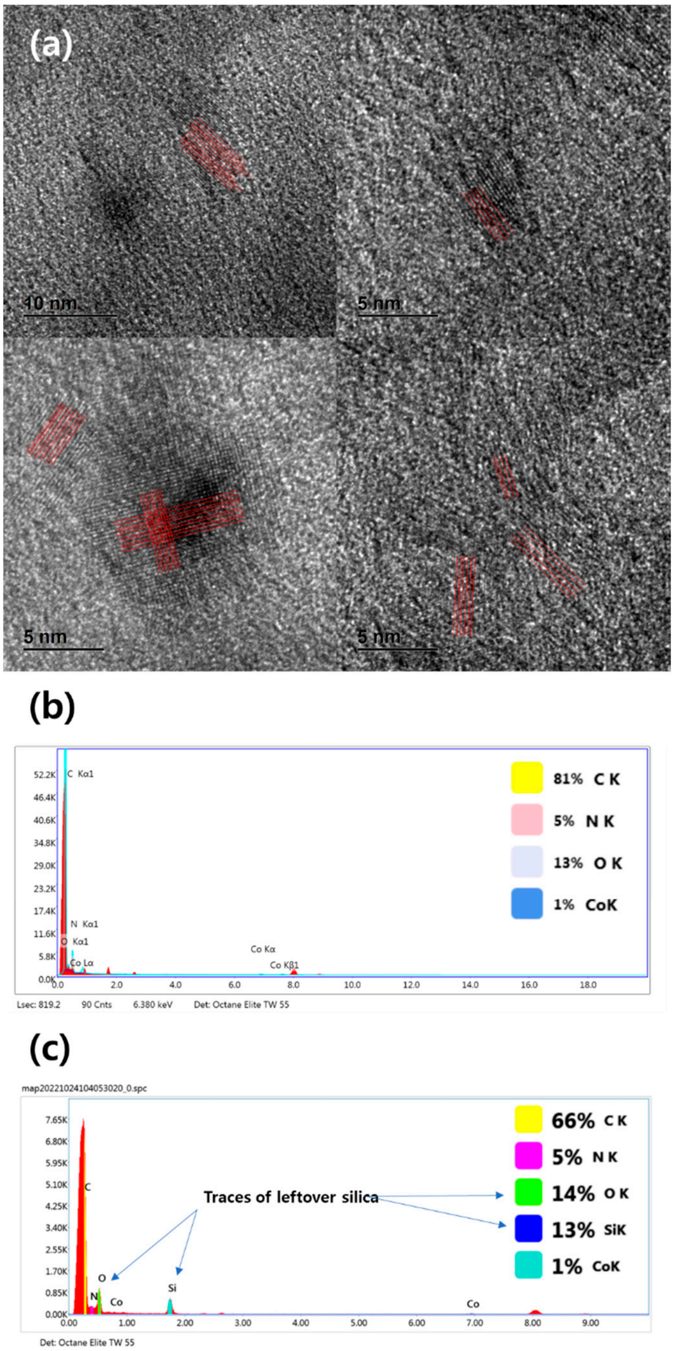

Figure S8. TEM analysis of the cobalt lattices of Co-N-HNCS. (a) Magnifications of spotted cobalt lattices with red lines for clarification. The sum spectrum of elemental mapping analysis for (b) Co-N-HNCS and (c) half-etched Co-N-HNCS.

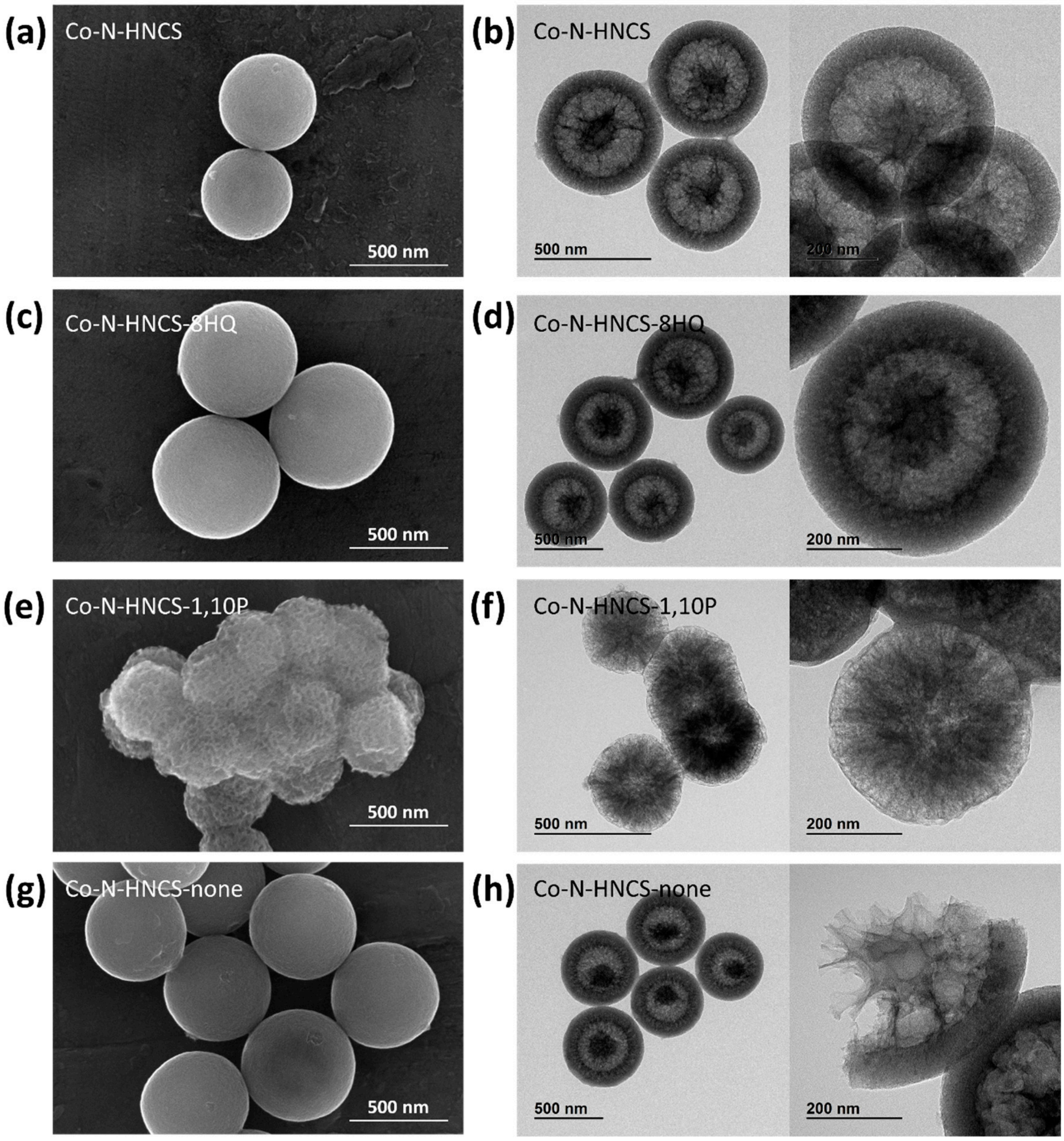

Figure S9. SEM and TEM images of variations of Co-N-HNCS. (a), (b) Co-N-HNCS, (c), (d) Co-N-HNCS-8 HQ, (e), (f) Co-N-HNCS-1,10 P, (g), (h) Co-N-HNCS-none.

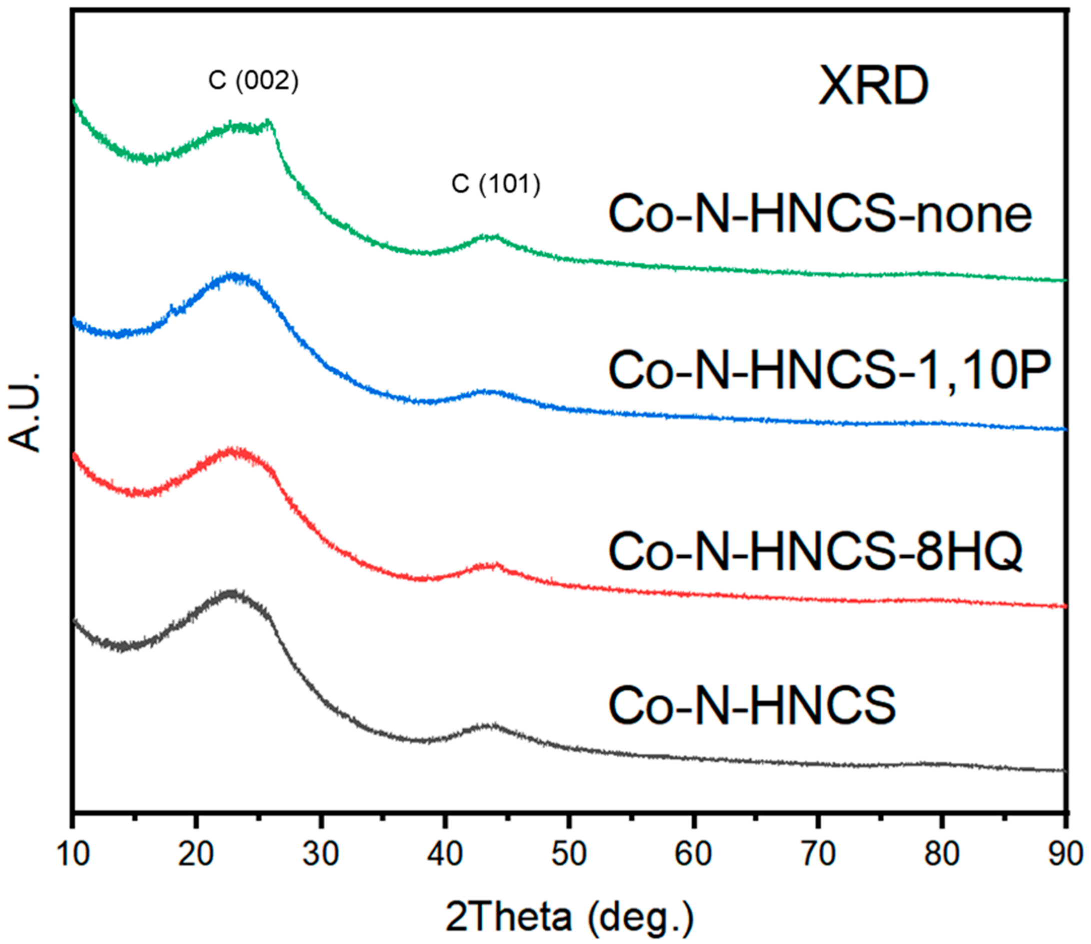

Figure S10. XRD patterns of Co-N-HNCS variation samples.

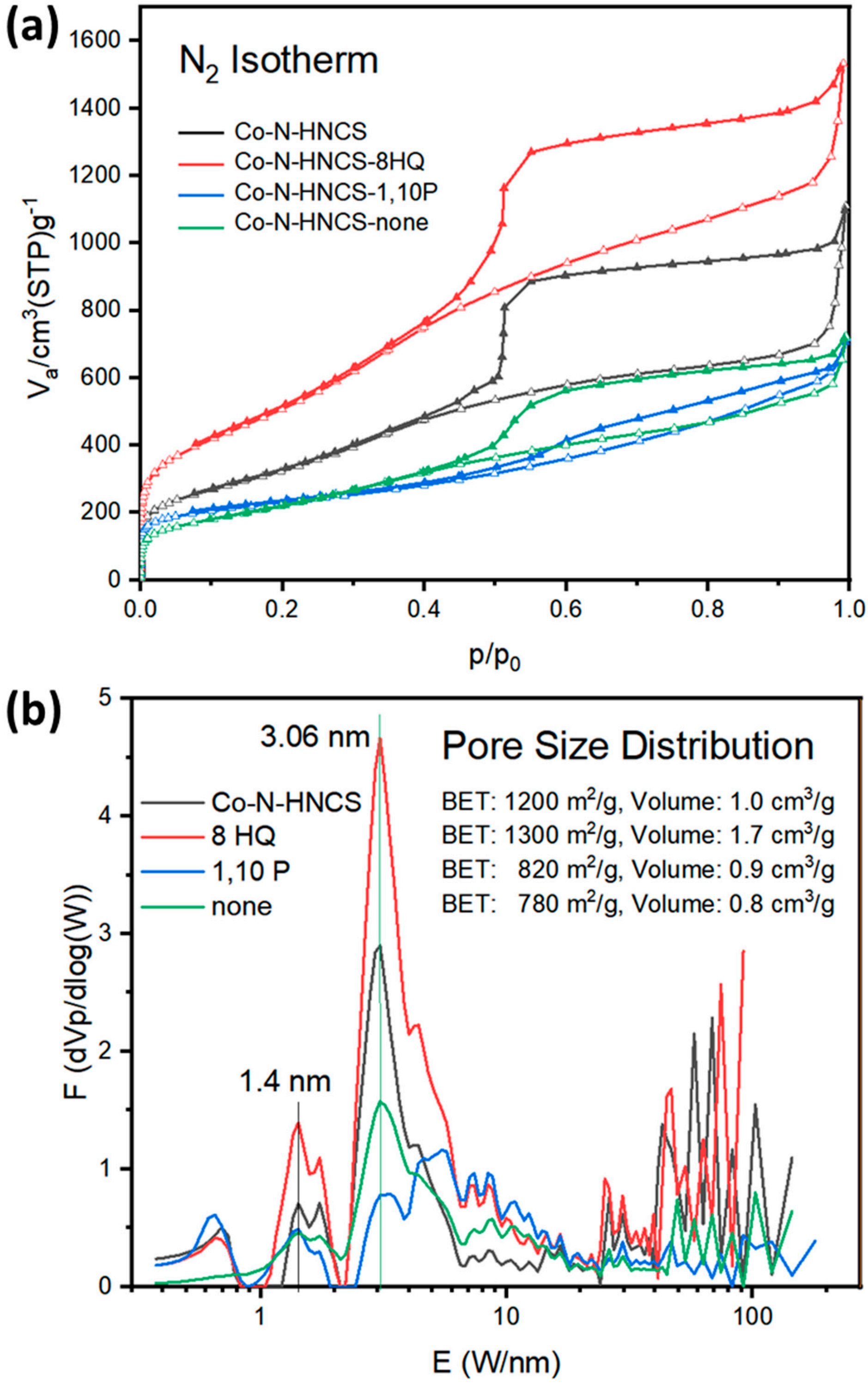

Figure S11. (a) Nitrogen adsorption isotherms and (b) Pore size distribution curves analyzed by NLDFT method of Co-N-HNCS variation samples.
